# IMPROVE: a feature model to predict neoepitope immunogenicity through broad-scale validation of T-cell recognition

**DOI:** 10.3389/fimmu.2024.1360281

**Published:** 2024-04-03

**Authors:** Annie Borch, Ibel Carri, Birkir Reynisson, Heli M. Garcia Alvarez, Kamilla K. Munk, Alessandro Montemurro, Nikolaj Pagh Kristensen, Siri A. Tvingsholm, Jeppe Sejerø Holm, Christina Heeke, Keith Henry Moss, Ulla Kring Hansen, Anna-Lisa Schaap-Johansen, Frederik Otzen Bagger, Vinicius Araujo Barbosa de Lima, Kristoffer S. Rohrberg, Samuel A. Funt, Marco Donia, Inge Marie Svane, Ulrik Lassen, Carolina Barra, Morten Nielsen, Sine Reker Hadrup

**Affiliations:** ^1^ Department of Health Technology, Technical University of Denmark, Lyngby, Denmark; ^2^ Instituto de Investigaciones Biotecnológicas, Universidad Nacional de San Martín, Buenos Aires, Argentina; ^3^ Center for Genomic Medicine, Copenhagen University Hospital, Copenhagen, Denmark; ^4^ Department of Oncology, Phase 1 Unit, Rigshospitalet, Copenhagen, Denmark; ^5^ Department of Medicine, Weill Cornell Medical College, New York, NY, United States; ^6^ National Center for Cancer Immune Therapy, Copenhagen University Hospital, Herlev, Denmark

**Keywords:** neoantigen, neoepitope prediction, machine learning, immunotherapy, immunoinformatics

## Abstract

**Background:**

Mutation-derived neoantigens are critical targets for tumor rejection in cancer immunotherapy, and better tools for neoepitope identification and prediction are needed to improve neoepitope targeting strategies. Computational tools have enabled the identification of patient-specific neoantigen candidates from sequencing data, but limited data availability has hindered their capacity to predict which of the many neoepitopes will most likely give rise to T cell recognition.

**Method:**

To address this, we make use of experimentally validated T cell recognition towards 17,500 neoepitope candidates, with 467 being T cell recognized, across 70 cancer patients undergoing immunotherapy.

**Results:**

We evaluated 27 neoepitope characteristics, and created a random forest model, IMPROVE, to predict neoepitope immunogenicity. The presence of hydrophobic and aromatic residues in the peptide binding core were the most important features for predicting neoepitope immunogenicity.

**Conclusion:**

Overall, IMPROVE was found to significantly advance the identification of neoepitopes compared to other current methods.

## Introduction

To be immunogenic, neoantigens derived from somatic mutations must be sufficiently expressed, presented, and recognized by immune cells. In this context, specifically, CD8+ T cells play an essential role by recognizing fragments of the neoantigens, called neoepitopes. When sufficiently induced, such T-cell responses may lead to tumor regression.

It has been shown that the tumor mutational burden (TMB) and the neoantigen load are predictive of patients’ response to immunotherapies, such as checkpoint inhibition (CPI) (1–3). Personalized immunotherapies based on vaccination with neoantigens are under clinical development and have demonstrated effective neoepitope-directed T-cell responses as well as good tolerability (4). However, the development of therapeutic strategies targeting neoepitopes depends entirely on the capacity to predict which of the many mutational alterations accumulating in tumors give rise to T-cell recognition. With current bioinformatic tools, only 2%–6% of the predicted neopeptides are demonstrated to give rise to T-cell recognition in CPI-treated cancer patients (5–7). This number needs to be greatly improved to facilitate the successful clinical implementation of neoepitope-targeting strategies.

Various methods have been developed to predict patient-specific neoepitopes from DNA and RNA sequencing (RNAseq). These methods rely on detecting somatic mutations that generate neopeptides and predicting their binding to the patient’s major histocompatibility complexes (MHCs) to generate a list of neoepitope candidates that could give rise to T-cell recognition in the given cancer patients (8–11). Improving the specificity of neoepitope detection is an area of intense investigation, and machine learning methods have been developed to rank neoepitope candidates in order to predict their potential of being immunogenic, but their predictive performance remains limited (8, 12). One of the main challenges for developing accurate neoepitope immunogenicity predictors is the limited available data that experimentally distinguish the immunogenic neoepitopes from the non-immunogenic neopeptides. The resource for experimental evaluation of neopeptide-specific T-cell responses is limited since such validation is laborious and expensive and requires a patient-specific peptide selection, and the breadth of peptides that can be evaluated is often limited by the scarce availability of patient biological material (13).

Another challenging aspect of neoantigen prediction is that the characteristics of each patient’s tumor and immune system will influence neoepitope immunogenicity uniquely in the individual patient. It is known that tumors evolve to be less immunogenic by the process of immunoediting (1, 7, 14). The downregulation of MHC transcription, the induction of T-cell exhaustion, and the modulation of the immune infiltrate by the production of different suppressor cytokines are some of the mechanisms that favor cancer progression and immune evasion. The introduction of immunotherapies in immunocompetent patients may shift the tumor microenvironment (TME) profile, stimulating neoantigen cross-presentation and enhancing T-cell activity, ultimately resulting in an effective antitumoral immune response (1). However, not all patients respond to immunotherapy, and even those that do may not take full advantage of the immunogenic potential of the neoantigens.

As a consequence, when screening patients for T-cell recognition toward neopeptides, such data will comprise a substantial number of false-negative data (i.e., neopeptides that have the potential to be immunogenic but are not recognized by the T cells in a given cancer patient) due to intrinsic immune and tumor characteristics. Such events form a false-negative data sink that challenges our predictive capacity. To compensate for this potential bias and improve the predictive capacity, it is necessary to also consider the association between neoantigen immunogenicity and the characteristics of the TME (15–18).

In this study, we explore the characteristics of immunogenic neoepitopes in order to improve their prediction. We have gathered a large dataset of more than 17,500 neoepitope candidates and screened for the presence of neoantigen reactive CD8+ T cells (NARTs) in 70 patients with different tumor types undergoing immunotherapeutic treatment. We applied barcoded MHC multimers to determine T-cell recognition of neoepitopes (13), hence distinguishing the immunogenic neoepitopes from the non-immunogenic neopeptides. Based on the described data, we developed a machine learning model, named IMPROVE, that integrates i) the neopeptide sequence; ii) neopeptide-derived features such as their physicochemical properties, the source mutation qualities, the likelihood of antigen presentation, and T-cell propensity; and iii) patient-specific tumor-derived features, including MHC expression in tumor cells, the cytolytic activity (CYT), and the different cell populations that constitute the TME. We found that the combination of these features increases the performance for the selection of immunogenic neoepitopes. This result suggests that the challenges in neoantigen prediction can be addressed by having sufficient available data and integrating multiple factors from the complex antitumor immune response.

## Results

### Selection of neopeptides and experimental evaluation of their immunogenicity

**Figure 1:**
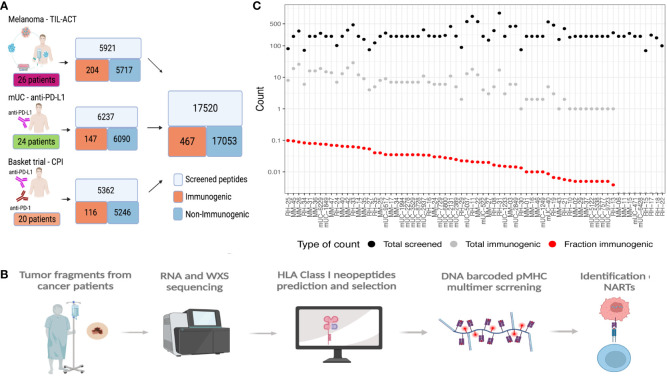
Data overview. **(A)** Data overview illustrating the number of validated peptides for each cohort and the number of patients screened together with a summary of the total amount validated with the number of immunogenic and non-immunogenic neopeptides. **(B)** General workflow of the data generation, including the patient samples being sequenced and patients’ specific libraries with neoepitope candidates being generated and screened with patients’ samples to find immunogenic neoepitopes. **(C)** Patient overview according to the number of neoepitopes screened (black dots), immunogenic neoepitopes (gray dots), and fraction immunogenic (red dots). MM, the melanoma cohort; mUC, the mUC cohort; RH, the basket trial cohort.

In this study, we have gathered experimentally validated neoepitopes from three different studies and assessed the features associated with the immunogenicity of predicted neoepitope candidates. We predicted all the neoepitopes from sequencing data of the tumors of 70 cancer patients, where each patient was experimentally evaluated for T-cell recognition covering a range of 100 to 1,092 neopeptides. Based on the screening for T-cell recognition using DNA barcode-labeled peptide–MHC multimers and holding each of these neopeptides, we identified T-cell responses against neoepitopes in the MHC I relevant for the given patient (19). Based on this experimental evaluation, we defined “immunogenic neoepitopes”, as those recognized by a T-cell population in at least one sample from the given patient, and “non-immunogenic”, as all neopeptides not recognized by T cells in our screen. In total, this study included 17,520 neopeptides, among which we found 467 (2.7%) to be recognized by a T-cell population in the corresponding patient (**Supplementary Table 1**), hence determined “immunogenic”. We generated these data through the screening of three patient cohorts, including different tumor types. One is a cohort of metastatic melanoma patients receiving adoptive cell transfer (ACT) with tumor-infiltrating lymphocyte (TIL) (TIL-ACT) (5). The second is a cohort of metastatic urothelial carcinoma (mUC) patients who received PD-L1 CPI (7). The third is a basket trial cohort with different cancer types and different CPI treatments (20) (**Figure 1A**). Their percentage of immunogenic neoepitopes found in the different cohorts was 3.45%, 2.36%, and 2.16%, respectively (**Supplementary Figure 1A**). We identified T-cell responses from either peripheral blood mononuclear cells (PBMCs) in mUC or PBMCs and TILs in the melanoma TIL-ACT and basket trial.

All neopeptides included for T-cell screening across the three studies were extracted based on the patient’s individual tumor mutational landscape, derived from paired tumor/normal whole-exome sequencing (WES) and RNAseq from each patient. Genome analysis tool kit (GATK) best practices (21) was applied to obtain somatic variants, followed by peptide extraction using MuPeXI (8) (**Figure 1B**). From this pool of neopeptides, on average, 250 (range 100–1,092) neopeptides were evaluated for T-cell recognition per patient. The predicted likelihood of MHC presentation of the neopeptides to each patient’s MHC was used as a selection criterion. The majority of the neopeptides included for T-cell screening were classified as binders [NetMHCpan 4.0 (22) and eluted ligand % Rank (RankEL) < 2] to the patient’s HLA class I molecules. Peptides from patients with a high number of candidate mutations were selected using a more restrictive HLA binding threshold (RankEL < 0.5), whereas the RankEL threshold was relaxed for patients with fewer neopeptide candidates to meet the inclusion of a minimum of 100 neopeptides per patient. The selection criteria resulted in a binding score distribution with two peaks (of strong and weak binders), as seen in **Supplementary Figure 1B**. Only neopeptides from transcribed regions [transcripts per million (TPM) >0.1] were included (**Supplementary Figure 1C**). In total, 36 different HLA class I molecules were covered for T-cell screening across all patients, and T-cell responses were found restricted toward 27 of these. Some HLA molecules obtained a significantly higher proportion of immunogenic neopeptides, including HLA-A0101, B1501, B4001, C0202, and C0602 (**Supplementary Figure 1D**). In general, no association was observed between the number of neopeptides included for T-cell screening and the number of immunogenic neoepitopes identified (**Figure 1C**). It should be noted that non-immunogenic peptides may hold properties related to immunogenicity and potentially give rise to T-cell recognition in other individuals or under different immunological circumstances than tested here, which represents an intrinsic challenge for defining “true” immunogenicity in the neoepitope space.

### Features influencing neoepitope immunogenicity

**Figure 2:**
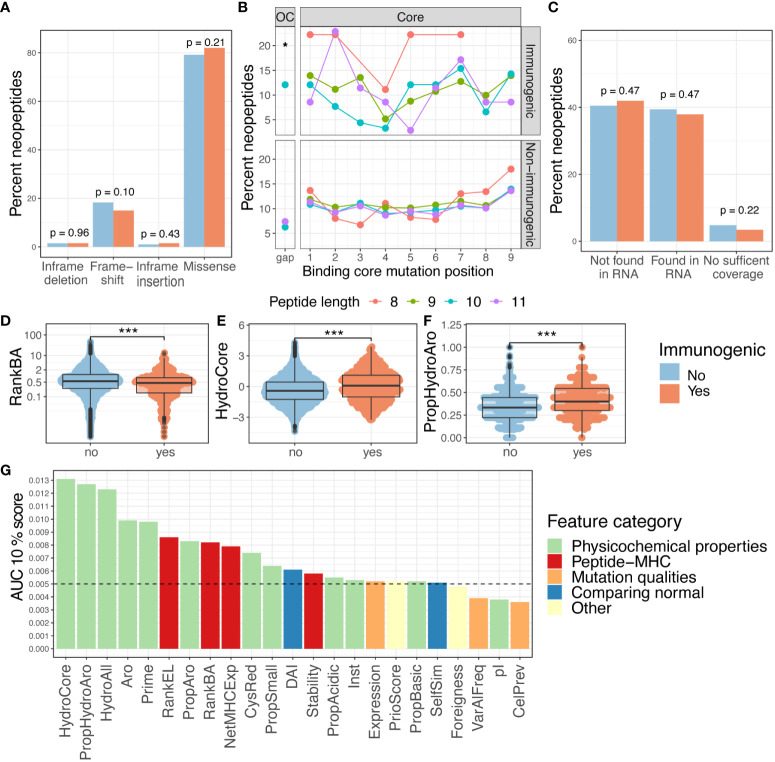
Features and immunogenicity. **(A)** Percentage of immunogenic neoepitopes according to the mutation consequence. The *p*-values were calculated according to the proportion test, testing if the number of immunogenic neoepitopes for each mutation type was present in a higher fraction compared to the non-immunogenic ones. **(B)** Fraction of immunogenic neoepitope for all missense mutations according to peptide position and peptide length. The gap position represents the peptide outside the core (OC) and is significantly enriched for neopeptides with a length of 10 (*p* = 0.01, prop.test). The neopeptides are separated into immunogenic and non-immunogenic neopeptides. **(C)** Percent of immunogenic and non-immunogenic neopeptides where the mutation was validated in RNA. A proportion test was performed to evaluate the proportion of immunogenic neoepitopes in the different categories. **(D–F)** Boxplot comparing the non-immunogenic form immunogenic neopeptides for four selected features; statistics by Wilcoxon test. **(D)** Peptide–MHC binding affinity (RankBA) *p* = 8.9·10^-9^. **(E)** Hydrophobicity only in the core of the peptide (HydroCore) *p* = 1.6·10^-12^. **(F)** Proportion of hydrophobic and aromatic residues in the peptide (PropHydroAro) *p* = < 2.22·10^-16^. **(G)** Performance with the partial AUC 10% for each feature with continuous values independently colored by feature type. p values < 0.05 = *; p values < 0.001 = ***.

First, a broad set of 27 individual features was interrogated, which have previously been hypothesized to influence neoepitope immunogenicity. Each feature was independently evaluated for its capacity to separate immunogenic *versus* non-immunogenic neoepitopes based on our compiled dataset. A total of 15 features demonstrated significance in separating the two categories of neopeptides (**Table 1**, **Figure 2**, **Supplementary Figure 2**).

**Table 1:** Feature overview.

Feature (abbreviation)	Description	*p*-Value
SelfSim	Self-similarity (mutant *vs.* normal peptide) (23)	*p* = 0.24
DAI	Differential agretopicity index (11)	*p* = 0.96
Mutation position	Position in peptide with mutation.	10-mer gap p= 0.01, prop test
Mutation consequence	The course of mutation	*p* = NS, prop test
CelPrev	Cellular prevalence (24)	*p* =0.016
Expression	Expression level (25)	*p* =0.16
VarAlFreq	Variant allele frequency (8, 26)	*p* =0.72
ValMutRNACoef	Validation of mutation in RNA sequencing	*p* = NS. (prop test and wilcox test)
Foreigness	Foreignness score (27, 28)	*p* = 0.24
PrioScore	Priority score (8)	*p* = 0.088
RankBA	Peptide–MHC binding with binding affinity % Rank (22)	*p* = 8.9·10^−9^
RankEL	Peptide–MHC binding with eluted ligand % Rank (22)	*p* = 0.0038
Stability	Peptide–MHC stability (29)	*p* = 0.012
NetMHCExp	NetMHCpanExp (30)	*p* = 0.15
PropHydroAro	Proportion of hydrophobic and aromatic residues (31)	*p* = < 2.22·10^−16^
Prime	PRIME score (32)	*p* = 9.3·10^−13^
HydroCore	Mean hydrophobicity in core (without anchor residues) (33)	*p* = 1.6·10^−12^
HydroAll	Mean hydrophobicity entire peptide (33)	*p* = 3.1·10^−12^
Aro	Aromaticity (31, 33)	*p* = 0.029
PropAro	Proportion of aromatic residues in non-anchor positions	*p* = 2.6·10^−09^
CysRed	Cysteine residues (31)	*p* = 1.5·10^−05^
PropSmall	Proportion of small amino acids in non-anchor positions	*p* = 0.003
PropAcidic	Proportion of acidic amino acids in in non-anchor positions	*p* = 0.003
Inst	Peptide instability (31)	*p* = 0.014
PropBasic	Proportion of basic amino acids in non-anchor positions of peptide	*p* = 0.088
pI	Isoelectric point (34)	*p* = 0.120
mw	Molecular weight (31)	*p* = 0.029

Feature abbreviation and a short description of the features. The p-values were all calculated using Wilcoxon test with Bonferroni-adjusted p-value in addition to the p-values specified by prop-test. The color code defines the feature category.

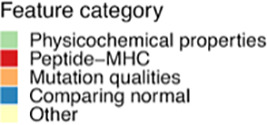

Of particular interest, whether the type and location of mutation influence immunogenicity was explored. The mutation types are categorized as missense, frameshift, in-frame insertion, and in-frame deletions. More immunogenic neopeptides were observed in the missense and frameshift categories, but these also constituted a larger fraction of the evaluated neopeptides; hence, no enrichment was observed (*p* = 0.1, proportion z-test) (**Figure 2A**). A previous study has reported the mutation position to be important for the immunogenicity of neoepitopes (35).

To investigate neopeptides of different lengths, we applied the predicted 9-mer binding core derived from NetMHCpan 4.1 (22) to all the neopeptides with missense mutations (36). By doing this, we uniformly investigated the role of the mutation position in the context of antigen presentation across neopeptides with variable lengths. In immunogenic neoepitopes, mutations were predominantly located around the anchor positions of MHC I motifs, while we observed fewer mutations near position 4 in 8-, 9-, and 10-mers and near position 5 in 11-mers (**Figure 2B**, top). We did not observe this pattern in non-immunogenic neopeptides (**Figure 2B**, bottom). We also observed a significantly increased frequency of mutations in the gap position (outside the core) in 10-mer neoepitopes (*p* = 0.04, proportion test) (**Figure 2B**) compared to the non-immunogenic ones, suggesting that mutations in longer peptides, in general, are facing out toward the T cells in immunogenic peptides, as gap positions are generally characterized by protruding residues.

As mutation calling can give false-positive mutation assignments (37–39), the presence of the WES-called mutation in the transcriptome was investigated. Among the predicted neopeptides, 47% of neopeptides originated from mutations that could be detected in at least one transcript of the RNAseq, 47% were not found in RNAseq, and 6% had insufficient RNA coverage in the region of the mutation (see Materials and Methods). No significant difference was found with this validation in separating immunogenic neoepitopes from non-immunogenic ones (**Figure 2C**, **Supplementary Figure 2A**). Additionally, the proportion of the mutated transcript (ValMutRNACoef) was not associated with neopeptide immunogenicity (**Supplementary Figure 2B**). Six other features related to mutation quality were evaluated, but only cellular prevalence (CelPrev) was found to significantly contribute to immunogenicity (**Table 1**). Among peptide–MHC (pMHC)-related features, predicted binding affinity % Rank (NetMHCpan 4.0 and RankBA) (**Figure 2D**) and RankEL (**Supplementary Figure 2C**) significantly separated the immunogenic from non-immunogenic neopeptides (**Table 1**). Thirteen different features describing the physiochemical properties were evaluated, and most of these also significantly separated the immunogenic from non-immunogenic neopeptides, for example, the mean hydrophobicity in non-anchor subsequence of the neopeptide (HydroCore) (**Figure 2E**) and “PropHydroAro”, a parameter that describes the proportion of hydrophobic and aromatic residues in the peptide (**Figure 2F**) (**Table 1**, **Supplementary Figure 2C**). Likewise, HydroCore and PropHydroAro were evaluated for the corresponding wt peptide, and it was observed that the wt sequences also differed in relation to these features when classified as “immunogenic” and “non-immunogenic” based on the properties of the corresponding neopeptides. The difference was less pronounced within the wt peptides, suggesting that immunogenic neopeptides hold mutations that further strengthen these characteristics compared to the wt sequence (**Supplementary Figures 2D, E**).

Tolerance affects T-cell immunogenicity, and therefore, the self-similarity of neoepitopes is a previously explored parameter. Neopeptides can be classified as “conserved binders” (CBs), with retained MHC presentation and mutation outside the MHC anchor positions, and “improved binders” (IBs), with mutations in the MHC anchor position leading to improved MHC binding. It has previously been suggested that primarily the CBs will be affected by tolerance since the wt sequence can also be presented. We evaluated self-similarity for all peptide categories and observed a lower self-similarity for the CBs than the IBs (**Supplementary Figure 2F**). However, the self-similarity does not significantly differ between the immunogenic from non-immunogenic neopeptides either when observing all peptides (**Supplementary Figure 2B**) or when considering the IBs and CBs individually (**Supplementary Figure 2G**).

In summary, more than one-half of the features (17/29) showed a significant difference between immunogenic and non-immunogenic neoepitopes. To assess the performance of these features to independently drive improved identification of immunogenic neoepitopes, the area under the receiver operating characteristic curve (AUC) and the partial area under the receiver operating characteristic (ROC) curve were calculated at 0.1 (AUC01). This latter metric was included to focus on the high specificity part of the ROC curve. Each feature independently reached a max performance of AUC01 = 0.013 and AUC = 0.62 (**Figure 2G**, **Supplementary Figure 2H**). This indicates that even though a significant difference was observed for a feature in differentiating immunogenic and non-immunogenic neopeptides (**Figures 2D–F**, **Supplementary Figure 2C**), a limited performance was obtained when evaluating AUC.

### Improved prediction of immunogenicity by random forest modeling

**Figure 3:**
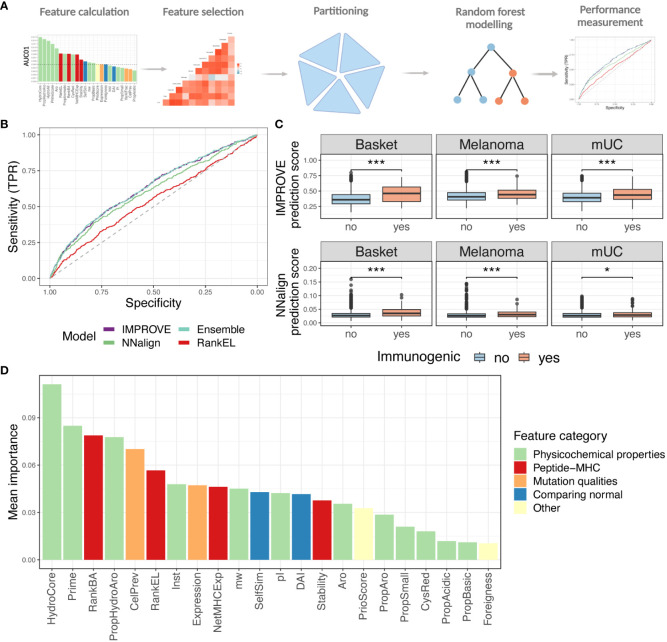
Random forest modeling. **(A)** Strategy of the machine learning approach with feature selection, partitioning, and modeling. **(B)** ROC curve with the IMPROVE model in purple (AUC = 0.630 and AUC01 = 0.0139), which performs significantly better than the NNAlign in green (AUC = 0.605 and AUC01 = 0.0131) (*p* = 0.039, roc.test) and RankEL (AUC = 0.539 and AUC01 = 0.0086) (*p* = 4.3^-6^). An Ensemble model of NNAlign and IMPROVE was also made, resulting in a similar performance as IMPROVE (0.631 and AUC01 = 0.0139), marked in a light blue line. **(C)** Prediction score from the NNAlign model at the top and IMPROVE model at the bottom according to the immunogenic and non-immunogenic peptide split by cohort. The IMPROVE model had significant separation in all three cohorts, with *p*-values of 1.6^-9^, 2.3^-6^, and 7.1^-6^ for the three cohorts. All with non-paired Wilcoxon test. The NNAlign model obtained significant separation in basket trial (*p* = 1.0^-10^, Wilcoxon test) and melanoma (*p* = 3.8^-7^, Wilcoxon test) and for the mUC cohort (*p* = 0.019). **(D)** Mean feature importance for the IMPROVE model colored by the feature category. p values < 0.05 = *; p values < 0.001 = ***.

To account for the joint influence of different features, random forest (RF) modeling was applied to develop the IMPROVE model, which predicts immunogenicity in our dataset, based on all the 27 features previously described. The overall workflow is illustrated in **Figure 3A**. Considering that previous studies have demonstrated that highly correlated features reduce the trainability of RF models (40, 41), the feature space was reduced to only include features with a mutual Spearman’s correlation coefficient lower than 0.7 (higher than −0.7 if negative). The feature selection was performed based on training in the fivefold cross-validation. The performance of the model was similar when performing the feature selection based on the entire dataset and deselecting the correlated feature with the lowest performance. This resulted in discarding HydroAll (correlated with HydroCore) and VarAlFrac (correlated with PriorScore) (**Supplementary Figure 3A**), and to simplify the model, the VarAlFrac and HydroAll were deselected for the fivefold cross-validation model. Furthermore, the binary one-hot-encoded features (i.e., yes/no features), including mutation consequence and mutation position, did not add predictive power (based on a backward feature selection) and therefore were not included in the model. Different models with backward and forward feature selection were evaluated to ensure that the final model gained the highest performance (data not shown). Based on these selection criteria, 22 features were incorporated into the final RF feature-based IMPROVE model. As an alternative strategy to the feature-driven model, a sequence-based model was also developed using the NNAlign method, which only encounters the peptide sequence (42).

A fivefold cross-validation scheme was used to train and evaluate both types of models. To avoid data redundancy, a modified common motif clustering was applied (described in Materials and Methods) to define the data partitions. This ensures that all the data from the same patient are partitioned together to avoid overfitting on patient-level features. The result was a dataset split into five partitions, separated by neoepitope candidate, common motifs, and patients.

The performance of the models was evaluated in terms of AUC and AUC01. The IMPROVE model displayed significantly higher performance (AUC = 0.630 and AUC01 = 0.0139) than NNAlign (AUC = 0.605 and AUC01 = 0.0131) (*p=*0.039), but both outperformed RankEL (AUC = 0.539 and AUC01 = 0.0086) (IMPROVE *vs.* RankEL *p* = 4.3^-6^) (**Figure 3B**). Similar results were obtained when using a precision–recall curve measurement. Also, here, IMPROVE was found to outperform both NNAlign and RankEL (**Supplementary Figure 3B**).

The performance of an ensemble model, based on the average score of both methods, demonstrated no major improvement with respect to the IMPROVE model alone (**Figure 3B**).

The separation of immunogenic and non-immunogenic neopeptides was further analyzed based on the prediction scores. For each cohort independently, a significant separation was observed using the prediction score from the IMPROVE model and NNAlign model. However, a clearer separation was found with the IMPROVE model, especially in the mUC cohort (**Figure 3C**). Both IMPROVE and NNAlign allow interpretation of the rules learned by trained models. Investigating the sequence logos produced by NNAlign, the immunogenic neopeptides were found to be enriched in hydrophobic and aromatic residues (**Supplementary Figure 3C**).

Also, when analyzing the feature importance for the IMPROVE model, the mean hydrophobicity and PRIME score (32) were found to be the most relevant features (**Figure 3D**).

In general, the prediction scores of the IMPROVE model were able to separate the immunogenic neoepitopes and non-immunogenic neopeptides also when evaluated for HLA alleles individually (**Supplementary Figure 3D, Supplementary Table 2**) and patients individually (**Supplementary Figures 3E–G**).

Neoepitopes are most often private antigens since most mutations are unique to the given patient’s tumor. However, given the size of the dataset in this study, we were able to detect several identical neopeptides present in the tumors of multiple patients. Specifically, 3% of the dataset corresponds to neopeptides whose sequence was identified in more than one patient and 2% of pMHCs that are present in more than one patient, as few shared neoepitopes are presented on different HLA alleles in different patients, despite the same peptide sequence. Following T-cell screening, we found T-cell recognition in three pMHCs presenting such “shared” neoepitopes. Interestingly, we found these neoepitopes to be immunogenic in only one patient and negative in one or two other patients, suggesting that patient-specific characteristics are influencing the neoantigen-directed immune response. This observation highlights the concern of a false-negative data sink, including non-immunogenic neopeptides that could have been immunogenic in another patient.

### Encountering tumor microenvironment improves prediction but not on patient level

The TME comprises an essential factor in the antitumoral immune response. Earlier studies have suggested that a combination of TMB and TME can be used as a biomarker to predict the patient’s response to immunotherapy (43–45). Also, it has been demonstrated that TME features influence the presence of TILs recognizing neoantigens (5). Immunogenic neoepitopes will only lead to tumor elimination if active neoantigen-reactive T cells are present. Based on RNAseq data, we derived information regarding the proportion of the different cell populations that compose the TME with the microenvironment cell population counter (MCP-counter) (15). Eight out of 10 of the populations from the MCP-counter significantly separated immunogenic from non-immunogenic neoepitopes and the last two (neutrophils and NK cells were non-significant) (**Supplementary Figure 4A**). We found a correlation between several of the cell populations (**Supplementary Figure 4B**), and consequently, due to the inability of the random forest model to detect the importance of highly correlated features, we calculated the mean of all cell estimates per patient (MCPmean). The MCPmean covers an estimate of the total immune cell infiltration.

**Figure 4:**
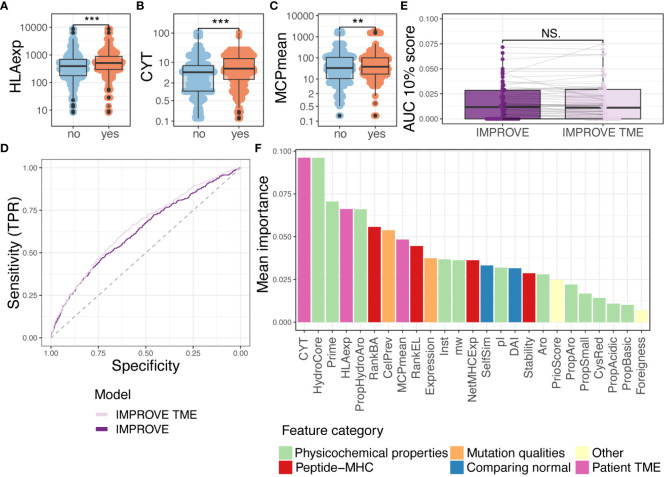
Random forest with tumor microenvironment (TME) parameters. **(A–C)** Comparing immunogenic with non-immunogenic neoepitopes for features. Statistics made using Wilcoxon test and Bonferroni-adjusted *p*-values. **(A)** HLA expression (HLAexp) *p* = 2.7^-15^. **(B)** Cytolytic activity (CYT) *p* = 9.3^-10^. **(C)** Mean of MCP-counter populations (MCPmean) *p* = 0.0075. **(D)** ROC curve illustrating the two IMPROVE models. The IMPROVE model without TME features in dark purple (AUC = 0.630 and AUC01 = 0.0139) and IMPROVE with TME features in light purple (AUC = 0.652 and AUC01 = 0.0145). IMPROVE TME is significantly better than IMPROVE (*p* = 0.01, roc.test). **(E)** The partial AUC 10% per patient for the two models and statistics made using paired Wilcoxon test (*p* = 0.95). **(F)** Mean feature importance for the IMPROVE with TME features colored by the feature type. p values < 0.01 = **; p values < 0.001 = ***; p-values > 0.5 = NS.

CYT, geometric mean of GZMA, and PRF1 expression can be used as estimates for T-cell cytolytic activity and have been found to correlate with the neoantigen load (46). Additionally, HLA presentation is an essential factor for neoantigen-directed T cell-mediated killing of cancer cells. Downregulation of HLA molecules is a known escape mechanism of tumor cells (47, 48), and the HLA expression in tumors correlates to higher immune cell infiltration and prolonged survival (1, 49). These TME features were found to significantly separate immunogenic to non-immunogenic neoepitopes (**Figures 4A–C**), and the separation in the HLA expression accounts for all HLA loci (**Supplementary Figure C**).

We integrated the patient-specific immune-related features in addition to the above-defined neopeptide-derived features in an extended IMPROVE model (IMPROVE TME) and checked that none of the TME features were correlated (**Supplementary Figure 4D**). Using the IMPROVE TME (including CYT, HLAexp, MCPmean, and previously described IMPROVE features), the performance of neoepitope detection significantly increased (*p* = 0.01) with a global AUC of 0.65 and partial AUC of 0.0145 (**Figure 4D**). We also observed the increased performance in precision–recall analysis (IMPROVE TME 0.052>0.049) (**Supplementary Figure 4E**). However, when observing performance per patient level, we did not see any increased performance (**Figure 4E**, **Supplementary Figure 4F**). To elaborate on this, we calculated the difference (delta) between the prediction scores derived from the IMPROVE TME and IMPROVE models (IMPROVE TME–IMPROVE). We observed that peptides from patients with high CYT had a positive delta, while peptides from patients with low CYT had a negative delta (Spearman’s correlation coefficient = 0.76 and 0.80) (**Supplementary Figure 4G**). In other words, the model with TME features favored peptides from patients with high CYT; therefore, TME features themselves were unable to distinguish the immunogenicity of peptides within patients but favored the patients with an immunocompetent TME. Hence, the improvement in AUC likely stems from the enrichment of T-cell responses in patients with favorable TME, which will also affect the immunogenicity classification in our dataset. Thus, to only determine peptide features associated with immunogenicity, the IMPROVE model can be used, but to include the likelihood of finding T-cell reactivity toward each neopeptide in each patient, the IMPROVE TME model should be applied.

Finally, we assessed the relative feature importance in the joint IMPROVE TME model. Here, CYT was the feature that constituted the highest importance for the model, whereas HLAexp was the fourth (**Figure 4F**). HydroCore and Prime were placed at second and third highest performance, respectively. In conclusion, TME and HLA expression are useful features to assess the proclivity of a patient to have a detectable T-cell response against immunogenic neoepitopes.

### Capturing of true-positive neoepitope and association with patient survival

**Figure 5:**
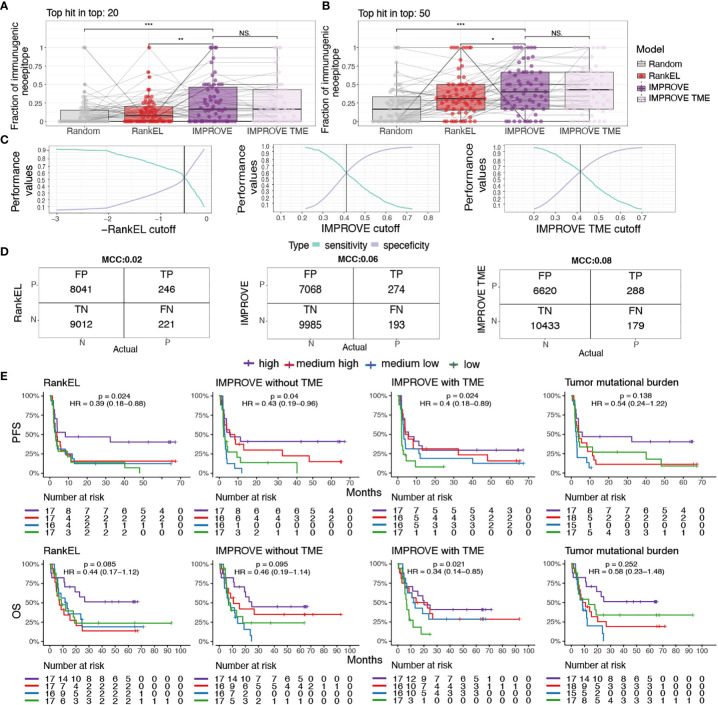
Patient performance and survival. **(A, B)** The fraction of immunogenic neoepitopes in the top 20 and top 50 neoepitope candidates of the IMPROVE model (dark purple) and the IMPROVE TME (light purple), with red indicating eluted ligand % Rank and gray indicating randomly sampled peptides. **(A)** Top 20 neopeptides IMPROVE *vs.* RankEL (*p* = 0.0023), IMPROVE *vs.* random (*p* = 8.2^-5^), and IMPROVE *vs.* IMPROVE TME (*p* = 0.64). **(B)** Top 50 neopeptides IMPROVE *vs.* RankEL (*p* = 0.03), IMPROVE *vs.* random (*p* = 1.8^-5^), and IMPROVE *vs.* IMPROVE TME (*p* = 0.71). **(C)** Sensitivity and specificity calculated for the cutoff where the point the curve crosses defines the set cutoff of what is predicted to be immunogenic and non-immunogenic. **(D)** Confusion matrix with cutoff where the sensitivity and specificity cross. The left image shows RankEL according to the pre-selected neoepitopes with expression above 0.01. The middle image shows the IMPROVE model without TME, and the confusion matrix on the right image is the IMPROVE model with TME features included, with the defined threshold found in panel **(C)**. **(E)** Kaplan–Meier curves showing all predicted neopeptides with a threshold of RankEL< 2 and Expression > 0.01, which included predicted neoepitopes that were not screened, for example, HLA alleles that were not available and neopeptides for patients selected with a more restricted threshold. The survival analysis was made for the three categories described in the confusion matrix. The patients were separated into four groups according to the number of predicted neoepitopes above the defined threshold. The four groups were determined according to the quantile, where “high” is above the third quantile, and “medium high” is between the second and third quantiles. “Medium low” is between the second and first quantiles, and low is below the first quantile. The threshold for predicted neoepitopes was set to where the sensitivity and specificity cross as shown in panel **(C)** and was also the threshold used in the confusion matrix. (Left) RankEL. (Middle left) The IMPROVE model without TME. (Middle right) The IMPROVE model with TME. (Right) The tumor mutational burden (TMB). p values < 0.05 = *; p values < 0.01 = **; p values < 0.001 = ***; p-values > 0.5 = NS.

To develop therapeutic interventions targeting neoepitopes, it is crucial to precisely determine relevant immunogenic neoepitopes. Hence, based on the IMPROVE prediction model, we investigated our capacity to catch true-positive immunogenic neoepitopes out of the total possible pool. We determined the proportion of observed immunogenic neoepitopes located among the top 20 and 50 ranked neopeptides per patient based on the predicted value from both the IMPROVE models (with and without TME), a RankEL prioritization, and random sampling. Both IMPROVE models showed a higher proportion of identified immunogenic neoepitopes out of the true immunogenic neoepitopes per patient compared to RankEL and random sampling. As expected from the description above, the implementation of the TME did not demonstrate any improvement compared to the model without TME on the patient level (**Figures 5A, B**).

We further evaluated the predictive value of the models for the identification of true positives (i.e., immunogenic neoepitopes validated through experimental detection of T-cell responses) based on a cutoff for the selection of predicted positive neopeptides using our IMPROVE models and RankEL for comparison. We set the cutoff as the point where the sensitivity and specificity curves intersect, meaning the point where we do not compensate for either the sensitivity or the specificity (**Figure 5C**). With the intersect cutoff according to RankEL, we identified 8,287 predicted positive candidates, of which 246 were true positive, obtaining a Matthews correlation coefficient (MCC) at 0.02 and an accuracy of 0.53. Increased performance was gained with the IMPROVE model, resulting in 7,342 predicted positive candidates, of which 274 were true positive (MCC at 0.06 and accuracy of 0.59), and an additional increased performance with the IMPROVE TME model resulted in 6,908 candidates, of which 288 were true positive (MCC at 0.08 and accuracy of 0.61) (**Figure 5D**).

To investigate the improved prediction capacity that also influences the use of neoepitope load as a biomarker for clinical outcomes, we predicted the immunogenicity score for all possible neoepitopes for each patient included in the studies addressed here. Using the previously defined cutoffs for the IMPROVE models and RankEL (**Figure 5C**), we separated patients into four groups by the quantile related to the number of predicted neoepitopes using the three different models. Using the IMPROVE-based selected, we observed an improved separation of survival in the four quantile groups, compared to RankEL, where only the highest quantile group was distinct (**Figure 5E**). The included cohorts were very heterogeneous, including different cancer types and treatments, and hence somewhat differently influenced by the level of neoepitopes when evaluated individually (**Supplementary Figure 5**). In comparison to the predicted neoepitopes, the TMB, which previously has been used as a biomarker for clinical outcomes for various cancer types, does not significantly separate the patients related to clinical outcome and results in a weaker hazard ratio compared to the neoepitope prediction (**Supplementary Table 3**, **Figure 5E**).

### Benchmarking on independent dataset and comparison of other tools

**Figure 6:**
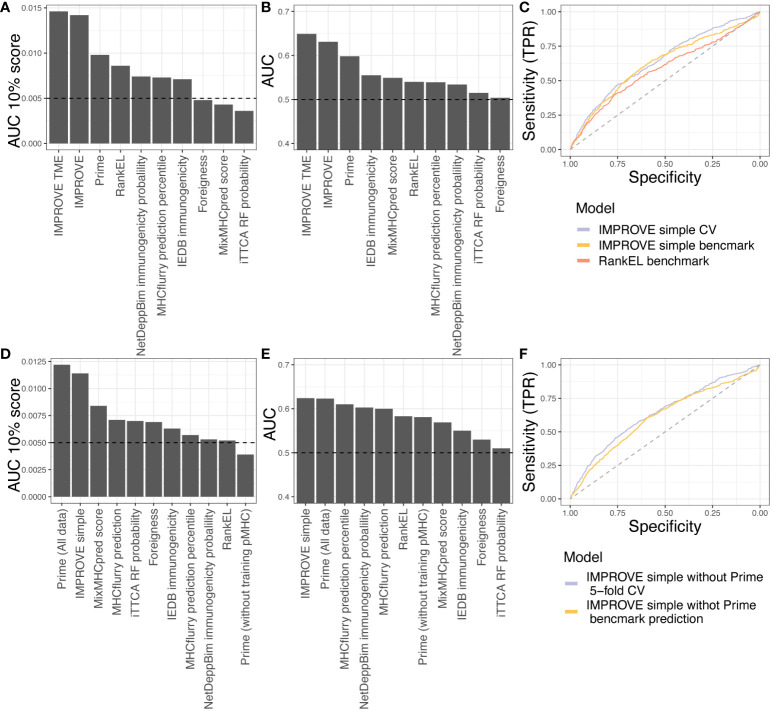
Benchmark data. **(A, B)** Testing the in-house dataset used to train IMPROVE with other available tools. **(A)** Performance according to the partial AUC 10%. **(B)** Performance according to AUC. **(C)** A simple IMPROVE model was generated using cross-validation, referred to as CV, taking features only by knowing the mutated peptide, corresponding WT peptide, and the HLA allele. This only excluded the Priority Score and cellular prevalence from the original IMPROVE model without TME. This IMPROVE simple model resulted in a performance of AUC = 0.643 and AUC01 = 0.0134 and is marked in light blue. The IMPROVE simple model to predict immunogenicity from the benchmark from CEDAR data performed only a bit worse than the IMPROVE simple model and is marked in yellow (AUC = 0.625 and AUC01 = 0.0102). The prediction of the CEDAR benchmark data using IMPROVE performed significantly better (*p* = 0.0038, roc.test) than RankEL as colored in red (AUC = 0.586 and AUC01 = 0.0094). **(D, E)** Testing the CEDAR dataset using other available tools. **(D)** Performance according to the partial AUC 10%. **(E)** Performance according to AUC. **(F)** Retraining of IMPROVE simple model without Prime feature (purple), resulting in AUC = 0.64 and AUC01 = 0.0135, and predicting CEDAR data with the IMPROVE simple model without Prime (yellow), resulting in AUC = 0.61 and AUC01 = 0.0104.

Next, we tested eight other publicly available tools’ predictive power over our in-house dataset. It should be noted that some of these tools have been included in the IMPROVE model (Foreigness, RankEl, and Prime). Some tools also have restrictions; for example, DeepNetBim only accepts 9-mer peptides, and therefore, the performance reported corresponds to different subsets where the models can be applied (see Materials and Methods). The tool with the best performance in addition to the IMPROVE model was Prime (AUC = 0.598 and AUC01 = 0.0098), which also is one of the features included in our model. In addition to IMPROVE or features included in IMPROVE, NetDeepBim had the best performance according to AUC01 (AUC01 = 0.0074), and IEDB had the best performance according to AUC (AUC = 0.555) (**Figures 6A, B**).

Finally, we evaluated the IMPROVE model performance with independent neoepitope data downloaded from the Cancer Epitope Database and Analysis Resource (CEDAR) (50). The query included positive and negative T-cell assays of neopeptides presented in human MHC class I. We did not include viral, germline, and self-antigens. We discarded neopeptides from the CEDAR with the exact same sequence as neopeptides from the IMPROVE model training dataset. We included only neopeptides of length 8 to 12 amino acids, where the reported cognate MHC has a four-digit resolution. Since the IMPROVE model was developed with prefiltered data, we only selected peptides from the CEDAR database with RankEL < 2. The final independent dataset consisted of a total of 2,436 neopeptides with 548 immunogenic neoepitopes. The genomic features included in PrioScore, i.e., the VarAlFreq, cellular prevalence, expression level, and TME features, were not available for this dataset. Only the expression level could be inferred with pepX (51). To overcome this issue, we created an IMPROVE “simple” model referred to as IMPROVE simple, excluding these missing features from the training data. The cross-validation performance of the IMPROVE simple model (AUC = 0.643 and AUC01 = 0.0134) was similar to that of the IMPROVE model (AUC = 0.630 and AUC01 = 0.0139). This aligns with the earlier analysis showing that peptide sequence features, such as hydrophobicity, were the most important performance features. Thereafter, we evaluated the IMPROVE simple model on the independent benchmark data from the CEDAR, resulting in a performance of AUC = 0.624 and AUC01 = 0.0114. This performance was significantly better (*p* = 0.009) than that of NetMHCpan RankEL alone (AUC = 0.583 and AUC01 = 0.0109) (**Figure 6C**). These results demonstrate that the IMPROVE model has predictive power beyond the data used for the model development.

Next, we compared the performance of IMPROVE to that of the eight other tools. We observed that IMPROVE was the best tool to predict immunogenic neoepitopes in the CEDAR dataset according to AUC and that Prime displayed the best performance when evaluated using AUC01 (**Figures 6D, E**). It should be noted that 70% of Prime’s training data were included in the CEDAR evaluation data, likely resulting in a performance overestimation from this method, confirmed by the finding that the PRIME performance dropped when removing the peptides overlapping with the PRIME training data from the CEDAR evaluation data, showing that Prime lacks performance with the independent dataset. To further investigate this, and since the PRIME prediction score is a feature of the IMPROVE model, we retrained the IMPROVE model without the Prime score feature. Doing this resulted in similar performance of the in-house dataset used for the fivefold cross-validation and a slight drop on the independent CEDAR dataset; however, it still has a better performance than the other tools in addition to the Prime with the whole dataset (**Figure 6F**). This demonstrates that IMPROVE is a state-of-the-art method for the accurate prediction of immunogenic neoepitopes, advancing the field of cancer antigen prediction.

## Discussion

Previous studies investigating neoepitope candidates have been hampered by limited data, making it challenging to learn the rules of their immunogenicity. In this study, we evaluated more than 17,000 neoepitope candidates with over 450 immunogenic neopeptides verified through large-scale T-cell interrogation using barcode-labeled peptide–MHC multimers (19). As such, this dataset is, to our knowledge, the largest experimentally verified pool of neoepitope candidates.

To overcome the scarcity of validated neoepitopes, other studies have predicted neoepitope immunogenicity based on pathogen-derived epitopes (28, 32), but the rules related to immunogenicity for pathogen-derived sequences may differ from those of neoepitopes, as they are potentially influenced by the tolerance to the wild-type sequence and the immune inhibitory microenvironment often established by the tumor. Consequently, these methods often have limited predictive performance on neoepitope data (12). A study that investigated immunogenicity characteristics based only on cancer neopeptides (including 30 immunogenic neoepitopes) found significant associations between immunogenicity and pMHC stability, affinity, and gene expression (11). Interestingly, in that study, hydrophobicity has the opposite selective characteristics compared to the results presented here. We found high hydrophobicity to be strongly associated with the immunogenicity of neoepitopes, which has also been demonstrated by others in relation to pathogen-derived epitopes (32, 52). This demonstrates the need for the assessment of larger neoepitope datasets, allowing data partitioning to avoid conclusions driven by few neoepitope examples.

In the present work, we interrogated 27 features covering mutation-specific, peptide sequence-specific, and patient-specific parameters. The large dataset of this study allowed us to accurately select the best features associated with T-cell responses and to develop a feature-based RF model (IMPROVE), providing insight into the most important characteristics of immunogenicity. Compared to other machine learning models, for example, artificial neural network (ANN) models, RF models are generally better at dealing with small datasets and numeric values in different scales and provide a greater understanding of the feature space (53, 54). From the RF modeling of our neoepitope dataset, we identified physicochemical properties of peptides relevant for their likelihood to be detected by T cells and specifically that immunogenic epitopes are enriched in hydrophobic and aromatic residues, supporting previous studies (32, 55). Next, the likelihood of binding to the MHC was found to be a relevant feature that influences immunogenicity, in line with previous studies (11, 28), but since our peptide library was preselected by the NetMHCpan score, the feature importance score does not accurately reflect the criticality of HLA binding for detecting neoepitopes. Prediction of binding to MHC is the most relevant step in neoepitope detection and should be addressed in all pipelines (56). Clonal characteristics of the mutation have previously been hypothesized as a feature contributing to immunogenicity (56)—here, we used cellular prevalence, a parameter related to mutational clonality, and observed this among the most essential features of the model, supporting that, indeed, mutational clonality influences immunogenicity. Lastly, features related to expression, self-similarity, pMHC stability, and foreignness provided a minor contribution to the final model.

In cancer patients, the TME, additionally, critically influences the ability to raise tumor-antigen-specific T-cell responses (47). Our study addressed the importance of TME features in a combination of prioritizing neoantigen candidates, and IMPROVE showed an increased performance by including these TME features in the prediction. Notably, the patient-specific TME features add information about patients but should not be applied alone because we did not observe an improved performance on the patient level when compared to the original model without TME features included. This underlines that the TME features select for patients that are more likely to mount a neoepitope T-cell response and therefore is useful for predicting the likelihood of T-cell response rather than prioritization of neoepitopes within a given patient.

From a large dataset, as presented here, sequence identity across neoepitopes can be investigated. This is represented by the NNAlign model, which demonstrates that such sequence-based training can identify dominant sequence characteristics related to hydrophobicity. However, as sequence characteristics may differ depending on HLA restriction, antigen sequence, etc., having a model built on epitope features is more likely to reveal characteristics of relevance across variable patient cohorts—and here, we demonstrated that feature-based models have a better predictive performance compared to more simple sequence-based models.

Previous studies showed that the level of T-cell recognition of neoantigens was associated with better clinical outcomes and survival (5, 6, 57). The mUC cohort showed a difference in survival, comparing the increase in neoepitope-specific T-cell responses after 3 weeks of treatment (6), and in the melanoma cohort, improved survival was demonstrated for patients with higher frequency and breadth of neoepitope recognition in their TIL infusion products (5). Importantly, we could demonstrate that the neoepitope load predicted using IMPROVE showed a higher correlation to patients’ overall survival compared to the neoepitope load predicted based on RankEL (using NetMHCpan) alone. Although the prediction of patient outcome is not the key accomplishment of the IMPROVE prediction strategy, the influence observed on patient outcome suggests that the predicted immunogenic neoepitopes are indeed relevant targets of T-cell recognition and elimination of cancer cells and do influence treatment outcome and survival.

The challenge for neoepitope prediction is the high level of false-negative data that will be embedded in every dataset. Identification and detection of neoepitope-specific T cells are challenging at a broad scale, and despite recent improvements in high-throughput strategies for pMHC-driven T-cell detection (5, 19), such strategies are still dependent on the quality and type of biomaterial available. Often, the frequency of neoepitope-specific T cells is very low, even below the detection limit; hence, a fraction of these can be missed during analyses. Tumor-infiltrating lymphocytes will in most cases hold larger frequencies of neoepitope reactive T cells compared to, e.g., peripheral blood (5), but TILs are rarely available, and as such, measuring of neoepitope reactive T cells in PBMCs may underestimate the number of neoepitopes that could, in fact, give rise to T-cell recognition. Furthermore, a large fraction of neopeptides may have the intrinsic characteristics to be immunogenic, but no T-cell response is mounted in the setting of the evaluation. This can relate to either the immune inhibitory environment of the tumor or the immunotherapeutic treatment given to the patients. It is demonstrated that immune-checkpoint inhibition does enhance the breadth of neoepitope recognition (6), but additional unleashed potential may be unexploited. Multiple studies have demonstrated that additional neoepitope response could be mounted upon antigen-specific stimulation, such as vaccination (58, 59). Thus, taken together, despite the size of our library, certainly not all potential immunogenic neoepitopes are captured, but the predictive value indicates that despite this limitation, the key features of immunogenicity can be captured.

The influence of each patient’s characteristics is further emphasized by the observation that the same pMHC complex can be immunogenic in one patient but negative when tested in others. Drawing a parallel to the investigation of epitopes from infectious diseases, several studies have demonstrated such variable behavior in, for instance, influenza (60) and SARS-CoV-2 (61) infections and can be explained by the phenomenon of immunodominance, differences in T-cell receptor (TCR) repertoire, and additional undefined patient characteristics. Thus, while this is a well-described phenomenon, it poses a substantial challenge to neoepitope data, as such epitopes are largely fully personalized, and hence, the immunogenicity of an individual neoepitope cannot be assessed by studying larger cohorts. In the field of pathogen research, a peptide would not be labeled as “negative” after testing only in one individual for immune response but rather would be evaluated based on the prevalence of T-cell recognition in a larger cohort. However, due to the private nature of neoepitopes, it is impossible to perform validation in multiple patients. This challenges the annotation of immunogenicity for neoepitopes, and to compensate for this limitation, large datasets are required to obtain accurate models for neoepitope prediction. The results and performance evaluations in the current and earlier epitope immunogenicity assessment/prediction work must be interpreted in the context of these limitations.

## Conclusion

This study introduces an improved model for the prediction of neoepitope immunogenicity, based on key selected features characterizing the neoepitopes. Such improvements are highly needed to advance the field of therapeutic neoepitope targeting. The IMPROVE model is built on a large dataset of neoepitopes evaluated for T-cell recognition in cancer patients. The model enhances the prediction of immunogenic neoepitopes and can identify true neoepitopes in more than 2/3 of the patients, within the top 20 neoepitope candidates. Furthermore, we raised the model performance by considering patients’ specific TME features. This can help to partially overcome the enormous patient variability that challenges the accuracy of neoepitope prediction. Even though the findings were based on broad-scale validated neopeptides, the model needs validation with more data, especially more immunogenic neoepitopes, to continue improving the performance. Nevertheless, the IMPROVE model can predict the ranking of neoepitope candidates with unprecedented accuracy, which is a critical task for the development of effective immunotherapeutic strategies targeting neoepitopes, like personalized cancer vaccines.

## Materials and methods

### Patient selection

The cancer patients included in this study were enrolled in three different studies: a melanoma cohort that received TIL-ACT (5, 62), a cohort of mUC patients who received PD-L1 checkpoint inhibition (6, 63), and a basket trial cohort with different cancer types and CPI treatments (20). Only patients with assessable patient material were included, which resulted in 70 patients in total.

### Neoepitope prediction

The neoepitope prediction was performed as described in Kristensen N et al. and Holm J et al. (5, 6). Shortly, paired tumor and normal WES and tumor RNAseq raw fastq files were pre-processed using trim-galore (64) version 0.4.0 to remove low-quality fragments and adapters. Burrows-Wheeler Aligner (65) version 0.7.15 was used to align the trimmed WES reads to the human reference genome (GRCh38), and MarkDuplicates from Picard-tools version 2.9.1 (66) was applied to tag duplicated reads due to technical artifacts. The quality scores were recalibrated using GATK, and the paired bam files were used as input to MuTect2 (26) from GATK (21) to detect somatic mutations. The bladder and melanoma cohort was processed following guidelines from GATK 3.8.0. GATK version 4.0.1 was used for the basket trial cohort. Tumoral transcript abundance was quantified using Kallisto version 0.42.1 (25) from RNAseq. Patient-specific HLA genotype was determined using OptiType (67) version 1.2 on WES derived from normal samples. HLA types, RNA expression, and somatic mutation VCF files were used as input to MuPeXI (8) version 1.1.2 to extract and rank neoepitope candidates. Posterior filtering of candidates was performed considering RNA expression values >0.01 and a predicted binding Rank <2 using NetMHCpan version 4.0 (22), except when patients had few peptide candidates, where the threshold was extended to 0.1 and RankEL < 0.5. If the expression level was insufficient to be obtained, they were reported as “-” in the MuPeXI output and were included in the peptide selection for the basket trial cohort. This covers in total 78 peptides from four patients.

### Neopeptide experimental immunogenicity assessment

In total, 17,520 neoepitope candidates were screened for their potential to activate a T-cell immune response with MHC multimer barcoding as described in References (5, 6, 13, 20). Predicted neopeptides and viral control peptides were synthesized using Pepscan (Pepscan Presto, Lelystad, The Netherlands). Peptides were dissolved to 10 mM in dimethyl sulfoxide (DMSO). Neopeptides were folded with the corresponding HLA and labeled with DNA barcodes, identifying all unique pMHC multimers. Each patient-specific multimer library was screened using patient-derived samples, including PBMCs and TILs, and the melanoma cohort was also screened using the infusion product used for the TIL-ACT.

### Feature calculation

#### Neopeptide features

The mutation consequence, as well as the sequences of mutant and wild-type peptides, were obtained using MuPeXI version 1.2 (8). The variant allele fraction (VarAlFrac) was obtained from mutect2, also given in the MuPeXI output as (Allele Frequency). The expression levels from the mutated transcript were calculated using Kallisto version 0.42.1 (25) and were obtained from the MuPeXI output. No transcript was assessable for 78 neoepitopes belonging to four patients from the basket trial, and in these cases, the expression was obtained from the Human Protein Atlas using NetMHCpanExp (30). The priority score (PrioScore) was also attained from the MuPeXI output. The likelihood of mutant and wild-type peptides binding to the patient’s MHC was predicted using NetMHCpan 4.0 (22).

Both the eluted ligand % Rank (RankEL) and binding affinity Rank (RankBA) predictions were retrieved. The differential agretopicity index (DAI) was calculated as the differences between the mutant and normal RankEL (Mutant Rank) (11). The stability of the pMHC complex was predicted using NetMHCstabpan 1.0 (29). Additionally, NetMHCpanExp-1.0 (NetMHCExp) was employed to jointly evaluate the expression of a peptide and its likelihood of binding to its cognate MHC and generating in this way a combined prediction that takes into account both features (30). Since anchor residues are in contact with MHC, non-anchor residues are more exposed to interaction with TCRs. Therefore, the non-anchor subsequence was defined as the fourth to the penultimate residue among the predicted binding core with NetMHCpan-4.1.

The foreignness score (Foreignness) was measured as previously described in Reference (27), and the function from antigen.garnish (28) was used to calculate the score. The similarity between the mutant peptide and the corresponding wild-type peptide or self-similarity (SelfSim) was calculated using the Kernel distance (23). Cellular prevalence (CelPrev) was calculated as previously described in Reference (5) using Sequenza (68) version 3.0, Shixiang/copy-number (69) version 1.26.0, and PyClone (24) version 0.13.1. Transcript abundance was derived from RNAseq data using Kallisto version 0.42.1 (25). To validate the expression of mutated alleles in RNA, the RNAseq was mapped to the reference genome using STAR v2.5.3 (70), and then all the bases were retrieved using samtools mpileup (71) at the variant positions reported by MuTect2. The proportion of mutated transcripts among the covered transcripts (ValMutRNACoef) was assessed using the formula Nreadssupporting variant/(Nreadsofcoverage+100) . Whether the mutation was present in the RNA in 15% of the neoepitopes was validated due to inconsistency in mutation position between MuPeXI and mutect2.

Physiochemical descriptors of the neopeptide sequences were calculated. The following were calculated using the ProteinAnalysis module from BioPython: molecular weight (mw); molar extinction coefficient; the relative frequency of F, W, and Y amino acids or aromaticity (Aro); instability index (Inst); and the relative frequency of V, I, Y, F, W, and L amino acids or helix (PropHydroAro) (31). The isoelectric point was calculated using EMBOSS with the Peptides package (34) in R. The mean hydrophobicity scale (33) and the proportion of different physicochemical classes of amino acids (small, aromatic, acidic, and basic) were calculated for the non-anchor subsequence. The propensity for TCR recognition was calculated using PRIME (32).

#### Patient features

The expression of MHC molecules (HLAexp) in the tumor cells was derived from RNAseq data. The CYT was calculated as the geometric mean of GZMA and PRF1, as previously described in Reference (46), and the expression of these genes was also derived from RNAseq data. The abundance of tumor-infiltrating immune and stromal cells was estimated with MCP-counter (15) using the RNAseq expression values obtained with Kallisto (25). MCPmean covers the mean abundance of all the 10 estimated cell populations from the MCP-counter including T-cells, CD8 T cells, Cytolytic Lymphocytes, NK cells, B linage, Monocyte linage, Myeloid dendritic cells, Neutrophils, Endothelial cells, and Fibroblasts.

### Models

#### Dataset

We assembled a dataset that contains the neopeptide sequences, the calculated features, and the immune response as the target value. For some neopeptides, it was not possible to obtain all the proposed features (there were no available RNAseq data for some patients, it was not possible to calculate the cellular prevalence when tumor samples were derived as cell lines, and MuPeXI does not report a normal peptide when a frameshift mutation has more than four mismatches compared to the most similar normal peptide, impeding the calculation of self-similarity). Therefore, we impute the missing values using the mean of the corresponding feature, except for the expression values in which we used NetMHCpanExp to retrieve expression values from the Human Protein Atlas (v. 20.0) database.

To avoid data leakage and overfitting of the models, a partitioning scheme was defined. A clustering algorithm was implemented to group neoepitopes based on i) shared subsequences or motifs between immunogenic neoepitopes and ii) the patient. In this way, all neoepitopes from the same patient and all similar neoepitopes were grouped together in the same partition. First, the immunogenic neoepitopes were grouped using the mentioned criteria, and then the non-immunogenic neopeptides were included in the defined partitions. If a peptide in the test set existed in the training data, the peptide was deselected from the training. This only affects the negative peptides, as the positive ones were separated by a partition.

#### Random forest

RF models were developed using the RandomForestClassifier module of the scikit-learn (72) package in Python version 3.7.6. The hyperparameter max_depth was set to 6, n_estimator was set to 2,000, min_sample_leaf was set to 6, and a nested cross-validation scheme was used. To avoid data imbalance, 500 negative data points (non-immunogenic peptide) were subsampled during training 50 times, and an ensemble score for the prediction was calculated.

#### NNAlign

The NNAlign version 2.1 (42) method was used to train a neopeptide sequence-based model with a fivefold nested cross-validation, using the same partition as the RF IMPROVE model. The motif length for the alignment was set to 6, and the flanking region was not considered. Like the IMPROVE model, if a peptide from the test set existed in training, the peptide was removed.

#### Survival analysis

Predicting survival probability with the effect of immunotherapy was based on the developed model from the study. The developed RF models were saved with “pickle dump” from pandas, and an ensemble score for each model was used as the immunogenicity score. The immunogenicity score was predicted for the remaining neopeptides with RankEL < 2 and expression > 0.01, which were not included in the study. This included, for example, peptides that were deselected for patients with many predicted neopeptides and therefore had stricter criteria during the neopeptide selection. Additionally, some HLA alleles were not assessable, and neopeptides with these alleles were also selected for immunogenicity screening. Patient RH-08 was excluded as the sample was taken after treatment, and MM-22 and MM-24 were excluded as the sample was from a cell line. The prediction score found for the remaining neopeptides was added on top of the peptides that were included in the study. This covers the dataset used for the survival analysis.

The separation of the four groups is based on the quantile of the variable. Patients with predicted neoepitopes above or equal to the third (75%) quantile were defined as “high”. Patients with peptides between the second (50%) and third quantile were defined as “medium high”, and patients with peptides between the first (25%) and second were defined as “medium low”. Lastly, patients below or equal to the first quantile were defined as “low”. The hazard score and *p*-value were calculated based on the comparison of the high *vs.* low group.

### Benchmark data

The benchmark data consisted of neoepitopes from the CEDAR and was filtered according to a RankEL < 2. This created a dataset of 2,436 peptides where 1,888 were categorized as non-immunogenic and 548 as immunogenic. The simple model to test the benchmark data has been developed in the same way as the RF IMPROVE model, but by excluding priority core and cellular prevalence. The expression level in the benchmark dataset was estimated using pepX (51).

### Immunogenicity prediction with other tools

DeepNetBim (73) only accepts peptides of nine amino acids. The predicted binding core with NetMHCpan 4.1 (74) was used to analyze all peptides included in the dataset. In 8-mers, the predicted position of insertion was replaced with X. DeepNetBim was downloaded and executed following the author’s recommendations. IEDB immunogenicity, MixMHCpred, and MHCflurry (75) were downloaded and executed locally following the author’s recommendations. iTTCA-RF (76) predictions were obtained from the corresponding web server.

### Statistics

All the statistics were calculated in R (77) version 4.1.1. The Wilcoxon rank-sum test was applied to analyze the features. Spearman’s correlation coefficients were used to measure the correlation between variables. To assess the performance of the models, the AUC and AUC01 were calculated using the function in ROCR version 1.0.11 (78), and the difference between ROC curves was computed using roc.test with default options from pROC version 1.18.0 (79). Kaplan–Meier curves were created using the survminer package version 0.4.9 (80), and the hazard ratios were calculated with the Cox proportional hazards regression model using the survival package version 3.3.1 (81).

## Data availability statement

All data and scripts used for the present manuscript can be found at: https://github.com/SRHgroup/IMPROVE_paper. To run IMPROVE and get an installation guide use the IMPROVE_TOOL page: https://github.com/SRHgroup/IMPROVE_tool.

## Ethics statement

The studies involving humans were approved by Capital region of Denmark + Institution Review Board of Memorial Sloan Kettering Cancer Center. The studies were conducted in accordance with the local legislation and institutional requirements. The participants provided their written informed consent to participate in this study.

## Author contributions

AB: Conceptualization, Data curation, Formal analysis, Investigation, Methodology, Software, Validation, Visualization, Writing – original draft, Writing – review & editing. IC: Data curation, Formal analysis, Investigation, Software, Validation, Writing – original draft, Writing – review & editing. BR: Formal analysis, Investigation, Software, Writing – review & editing. HG: Formal analysis, Software, Writing – review & editing. KKM: Formal analysis, Software, Supervision, Writing – review & editing. AM: Formal analysis, Software, Writing – review & editing. NP: Data curation, Resources, Validation, Writing – review & editing. ST: Data curation, Resources, Validation, Writing – review & editing. JH: Data curation, Resources, Validation, Writing – review & editing. CH: Data curation, Resources, Validation, Writing – review & editing. KHM: Data curation, Resources, Validation, Writing – review & editing. UH: Data curation, Resources, Validation, Writing – review & editing. ALS-J: Formal analysis, Software, Writing – review & editing. FB: Data curation, Resources, Writing – review & editing. VD: Data curation, Resources, Writing – review & editing. KR: Data curation, Resources, Writing – review & editing. SF: Data curation, Resources, Writing – review & editing. MD: Writing – review & editing. IS: Data curation, Resources, Writing – review & editing. UL: Data curation, Resources, Writing – review & editing. CB: Software, Supervision, Writing – review & editing. MN: Conceptualization, Formal analysis, Investigation, Methodology, Software, Supervision, Visualization, Writing – original draft, Writing – review & editing. SH: Conceptualization, Data curation, Funding acquisition, Investigation, Methodology, Project administration, Resources, Supervision, Validation, Visualization, Writing – original draft, Writing – review & editing.
