# Supplementary material for: IMPROVE: a feature model to predict neoepitope immunogenicity through broad-scale validation of T-cell recognition
Source: Front Immunol. 2024 Apr 3;15:1360281. doi: 10.3389/fimmu.2024.1360281 (PMC11021644; doi:10.3389/fimmu.2024.1360281)
Supplement: Supplementary file 1 [file DataSheet_1.pdf]

Supplementary Figures

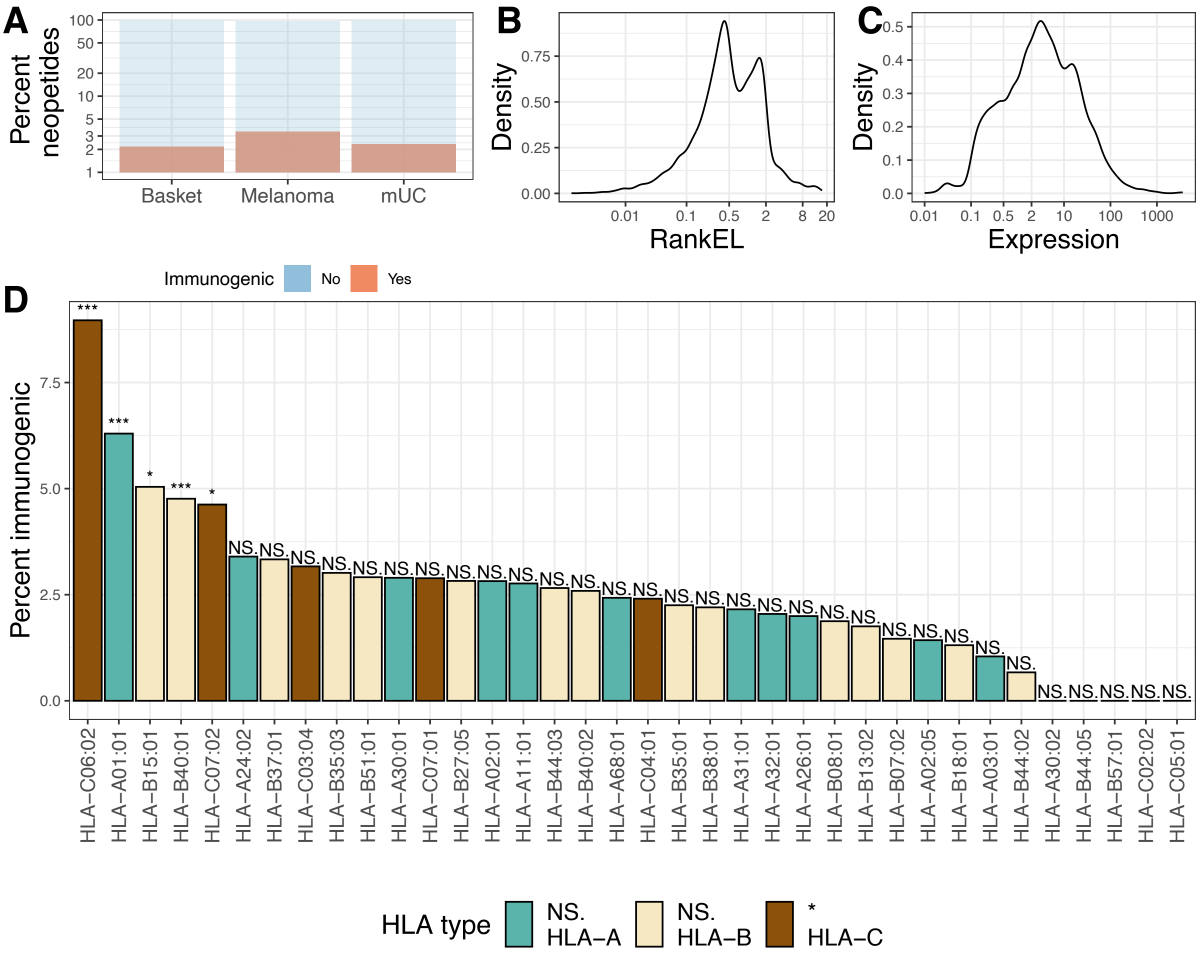

Supplementary Figure 1: Data overview. A) The percent of immunogenic and non-immunogenic neoepitopes within the three cohorts. B) Density of Eluted Ligand % Rank (RankEL) for all neoepitope candidates. C) Density of Expression level (Expression) from the corresponding genes for neoepitope candidates. D) The percent of immunogenic neoepitopes for each HLA allele colored by the HLA class test made by proportion z-test. p values < 0.001 = ***, p values < 0.01 = ** p values < 0.05 = * p-values > 0.5 = NS.

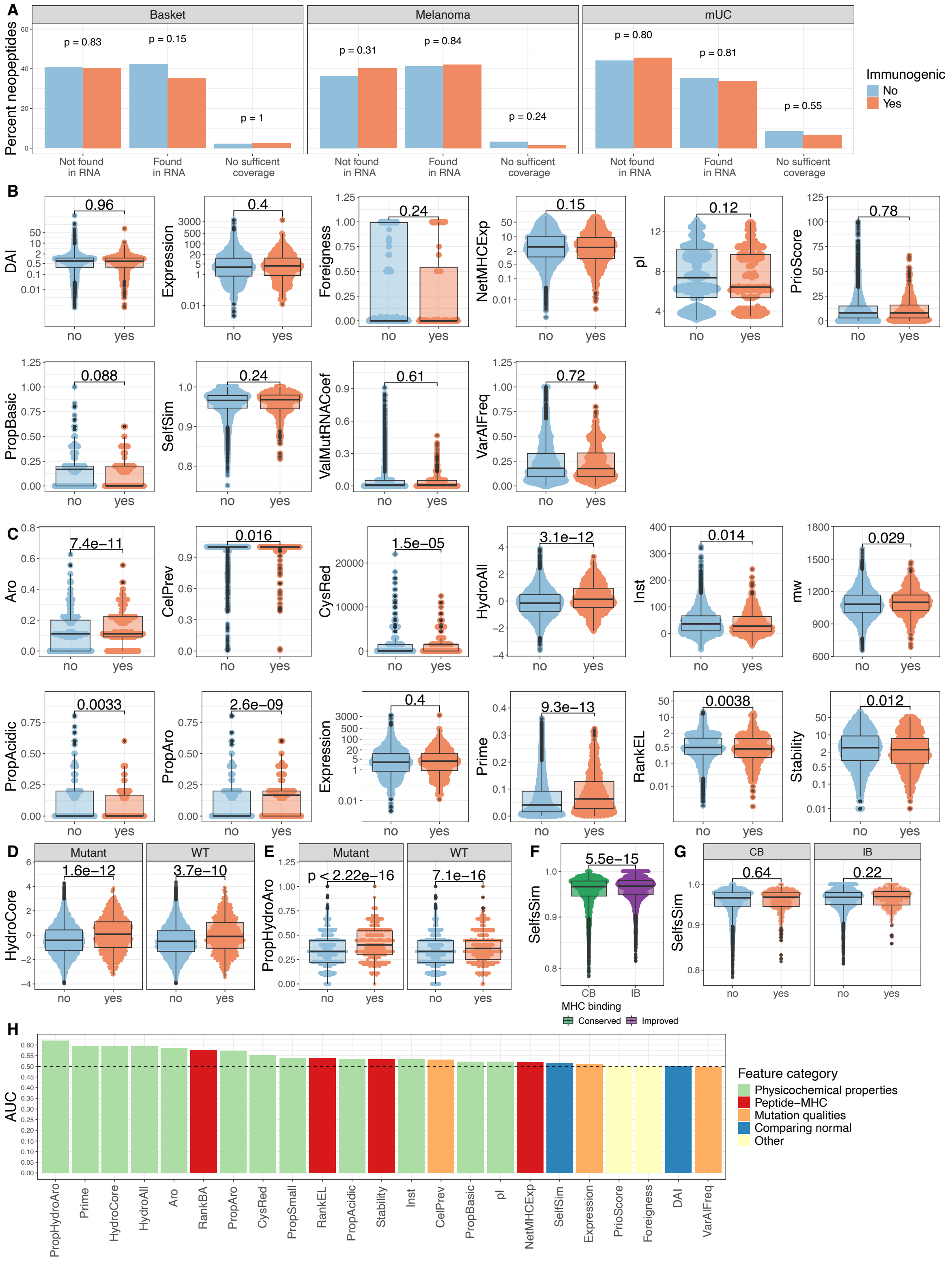

Supplementary Figure 2: Evaluation of individual features impacting immunogenicity. A) Validation of mutations in RNAseq for each cohort no significant difference according to a proportion test. B+C) Comparison of immunogenic and non-immunogenic neoepitopes according to the values of each feature. Statistics. Wilcox test with p-value adjustment by Bonferroni. B) Non-significant features, C) Significant features. D-E) Comparing immunogenicity from wild-type peptides according to HydroCore (D) and PropHydroAro (E), by considering the wild-type (wt) peptide immunogenic where the mutant peptide was found immunogenic. F) Self-similarity comparing Improved binder (IB) and conserved binder (CB). G) Self-similarity comparing immunogenic and non-immunogenic neoepitopes for each MHC-binding group (IB and CB). H) AUC for all features individually colored by feature type.

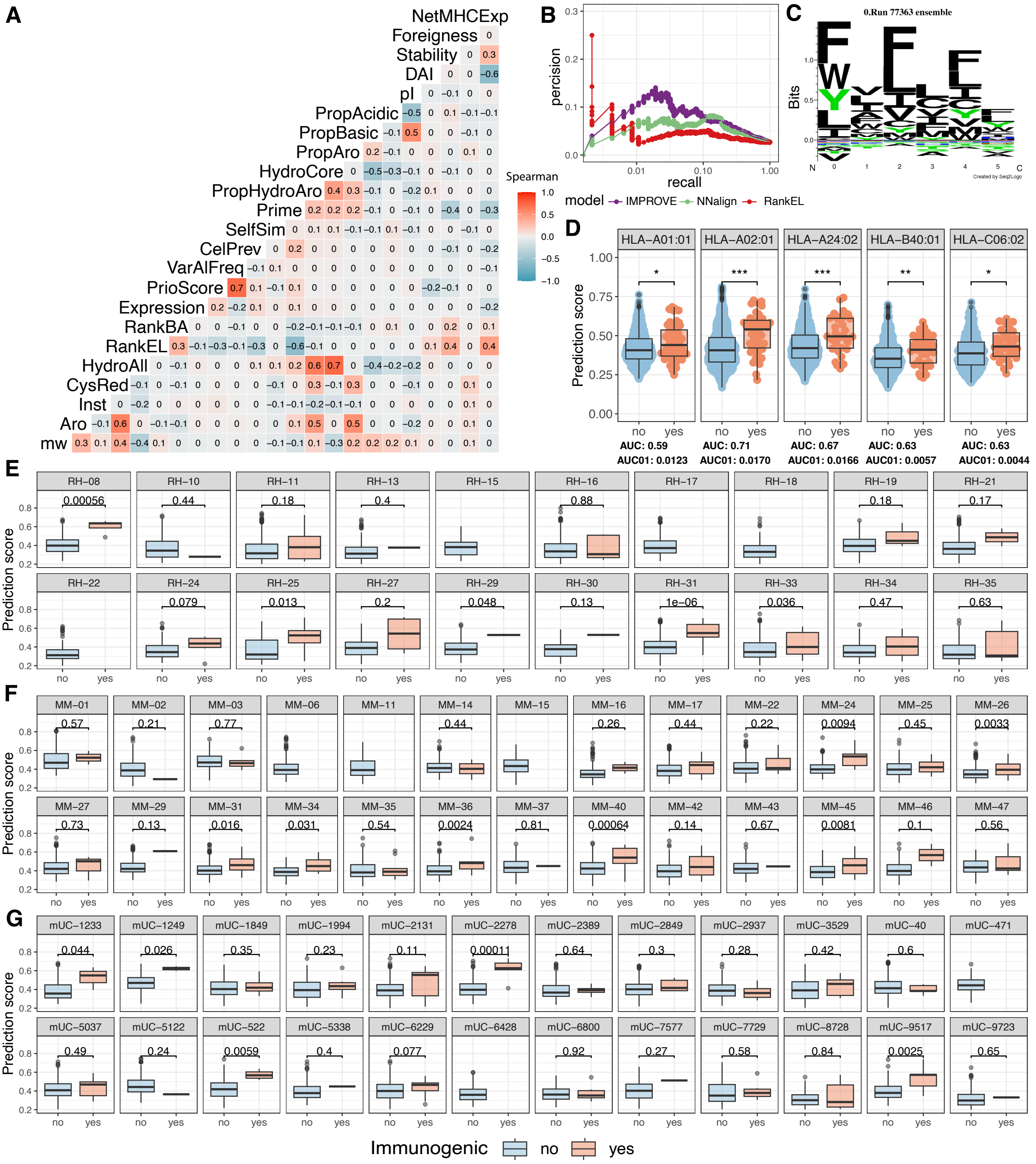

Supplementary Figure 3: IMPROVE modeling. A) Spearman correlation comparing all features. B) precision-recall curve for IMPROVE in purple with a pr-auc at 0.05, NNAlign in green) with a pr-auc at 0.04 and RankEL in red with a pr-auc at 0.03. C) NNAlign logo plot from 5-fold cross-validation. D) Performance for the five HLA-alleles with the most immunogenic neoepitopes. p-values: A01:01 = 0.03, A02:01=1.2^{-6}, A24:02 = 0.00093, B40:01 = 0.0068, C06:02 = 0.014. E-G) Comparison of the prediction score from the random forest model with the immunogenic and non-immunogenic neoepitopes for each patient in each cohort. p values calculated with the non-paired Wilcoxon test. E) Basket trial cohort, F) Melanoma cohort, G) mUC cohort. p values < 0.001 = ***, p values < 0.01 = ** p values < 0.05 = * p-values > 0.5 = NS

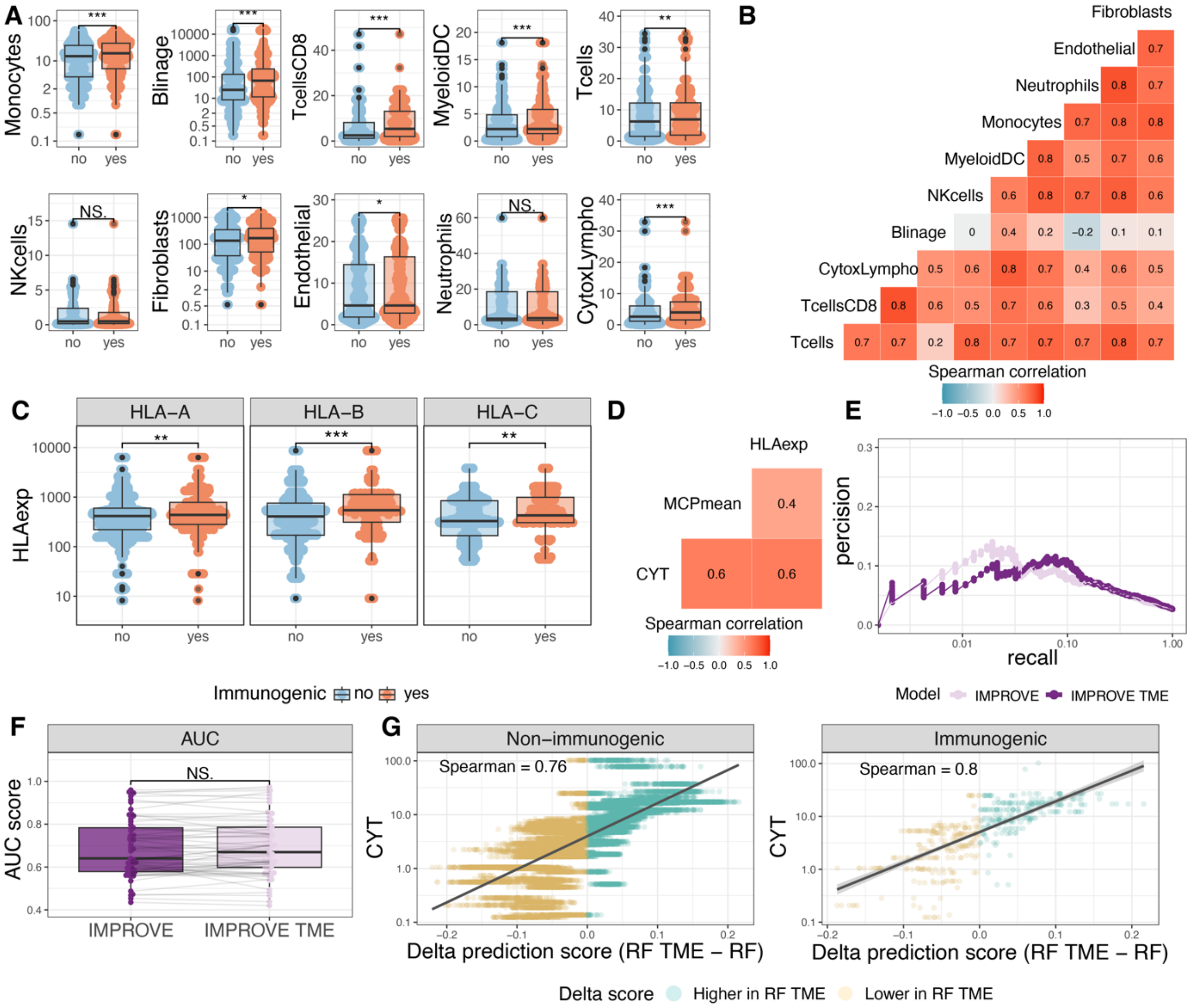

Supplementary Figure 4: random Forrest with Tumor Microenvironment (TME) features. A) MCP-counter cell populations comparing immunogenic and non-immunogenic neoepitopes. B) Spearman correlation of all the cell populations from MCP-counter. C) HLA expression per HLA class comparing immunogenic and non-immunogenic neoepitopes. HLA-A p = 0.0024, HLA-B p= 6.3^{-7}, HLA-C p = 0.006 (all with Wilcox test). D) Spearman correlation of all the TME features included in the IMPROVE TME model. E) Precision-recall curve for the IMPROVE (pr-auc =0.049) and IMPROVE TME model (pr-auc = 0.052). F) AUC per patient comparing IMPROVE and IMPROVE TME. G) Cytolytic Activity (CYT) on the y-axis and the delta prediction score calculated by the IMPROVE TME minus the IMPROVE score separated into immunogenic and non-immunogenic neopeptides. Correlation performed with spearman correlation. p values < 0.001 = ***, p values < 0.01 = ** p values < 0.05 = * p-values > 0.5 = NS

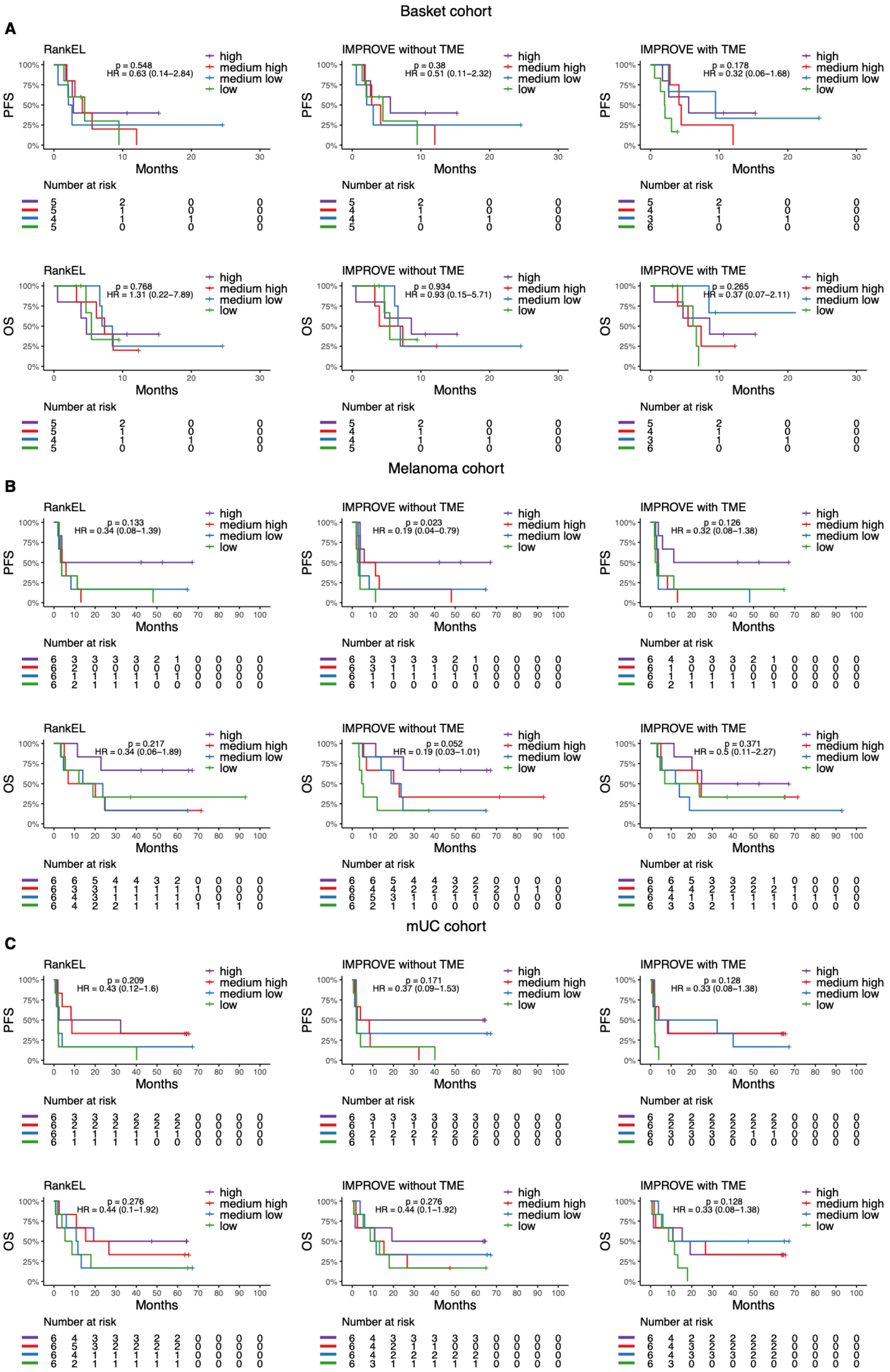
Basket cohort
A
RankEL
p = 0.548
HR = 0.63 (0.14–2.84)
IMPROVE without TME
p = 0.38
HR = 0.51 (0.11–2.32)
IMPROVE with TME
p = 0.178
HR = 0.32 (0.06–1.68)
high
medium high
medium low
low
PFS
Months
Number at risk
RankEL
p = 0.768
HR = 1.31 (0.22–7.89)
IMPROVE without TME
p = 0.934
HR = 0.93 (0.15–5.71)
IMPROVE with TME
p = 0.265
HR = 0.37 (0.07–2.11)
OS
Melanoma cohort
B
RankEL
p = 0.133
HR = 0.34 (0.08–1.39)
IMPROVE without TME
p = 0.023
HR = 0.19 (0.04–0.79)
IMPROVE with TME
p = 0.126
HR = 0.32 (0.08–1.38)
RankEL
p = 0.217
HR = 0.34 (0.06–1.89)
IMPROVE without TME
p = 0.052
HR = 0.19 (0.03–1.01)
IMPROVE with TME
p = 0.371
HR = 0.5 (0.11–2.27)
mUC cohort
C
RankEL
p = 0.209
HR = 0.43 (0.12–1.6)
IMPROVE without TME
p = 0.171
HR = 0.37 (0.09–1.53)
IMPROVE with TME
p = 0.128
HR = 0.33 (0.08–1.38)
RankEL
p = 0.276
HR = 0.44 (0.1–1.92)
IMPROVE without TME
p = 0.276
HR = 0.44 (0.1–1.92)
IMPROVE with TME
p = 0.128
HR = 0.33 (0.08–1.38)

Supplementary Figure 5: Survival curves per cohort. A-C) Kaplan-Meyer curves for the three categories (left) eluted ligand %Rank (RankEL), middle IMRPOVE model without TME features, and (right) the IMPROVE model including the TME features. Patients are separated into four groups according to the number of predicted neoepitopes above a defined threshold with Rank<2 and expression level > 0.01. The four groups are defined according to the quantile where "high" is above the 3rd quantile, "medium high" is between the 2nd and 3rd quantile. "medium low" is between the 2nd and 1st quantile, and low is below the 1st quantile. A) Basket trial cohort, B) melanoma cohort, C) mUC cohort.

Supplementary Tables

Supplementary Table 2: The number of immunogenic and non-immunogenic peptides and the corresponding performance for AUC and the partial AUC 10% (AUC01). Performance is calculated for all HLA alleles with 10 or more immunogenic neoepitopes.

HLA_allele	*non-immunogenic*	*Immunogenic*	*AUC*	*AUC01*
HLA-A01:01	744	50	0,5915	0,0123
HLA-A02:01	1516	44	0,7147	0,017
HLA-B40:01	760	38	0,6301	0,0057
HLA-C06:02	335	33	0,6299	0,0044
HLA-A24:02	881	31	0,6747	0,0166
HLA-B07:02	1752	26	0,6792	0,0268
HLA-C07:01	874	26	0,6348	0,0196
HLA-C07:02	433	21	0,5989	0,0155
HLA-B08:01	837	16	0,674	0,0238
HLA-C04:01	649	16	0,5482	0,0073
HLA-B38:01	666	15	0,6878	0,0219
HLA-C03:04	428	14	0,4624	0,0008
HLA-A03:01	1230	13	0,5214	0,0012
HLA-A31:01	590	13	0,5583	0,004
HLA-A11:01	422	12	0,7237	0,0211
HLA-B15:01	226	12	0,7367	0,0052
HLA-A26:01	540	11	0,8596	0,0274
HLA-B27:05	344	10	0,718	0,021
HLA-B35:01	434	10	0,6288	0,0071
HLA-A30:01	268	8	NA	NA
HLA-B44:03	293	8	NA	NA
HLA-A32:01	335	7	NA	NA
HLA-B35:03	193	6	NA	NA
HLA-B51:01	200	6	NA	NA
HLA-A68:01	201	5	NA	NA
HLA-B40:02	188	5	NA	NA
HLA-B44:02	593	4	NA	NA
HLA-B18:01	226	3	NA	NA
HLA-B13:02	112	2	NA	NA
HLA-A02:05	69	1	NA	NA
HLA-B37:01	29	1	NA	NA
HLA-A30:02	62	0	NA	NA
HLA-B44:05	45	0	NA	NA
HLA-B57:01	38	0	NA	NA
HLA-C02:02	515	0	NA	NA
HLA-C05:01	25	0	NA	NA
